# The Good, the Bad, the Question–*H19* in Hepatocellular Carcinoma

**DOI:** 10.3390/cancers12051261

**Published:** 2020-05-16

**Authors:** Lysann Tietze, Sonja M. Kessler

**Affiliations:** Institute of Pharmacy, Experimental Pharmacology for Natural Sciences, Martin Luther University Halle-Wittenberg, 06120 Halle (Saale), Germany; lysann.tietze@pharmazie.uni-halle.de

**Keywords:** H19, IGF2, miR-675, HCC, DMR, imprinted gene, 91H, HOTS, chemoresistance

## Abstract

Hepatocellular carcinoma (HCC), the most common primary liver cancer, is challenging to treat due to its typical late diagnosis, mostly at an advanced stage. Therefore, there is a particular need for research in diagnostic and prognostic biomarkers and therapeutic targets for HCC. The use of long noncoding (lnc) RNAs can widen the list of novel molecular targets improving cancer therapy. In hepatocarcinogenesis, the role of the lncRNA *H19*, which has been known for more than 30 years now, is still controversially discussed. *H19* was described to work either as a tumor suppressor in vitro and in vivo, or to have oncogenic features. This review attempts to survey the conflicting study results and tries to elucidate the potential reasons for the contrary findings, i.e., different methods, models, or readout parameters. This review encompasses in vitro and in vivo models as well as studies on human patient samples. Although the function of *H19* in HCC remains elusive, a short outlook summarizes some ideas of using the *H19* locus as a novel target for liver cancer therapy.

## 1. Introduction

Hepatocellular carcinoma (HCC) is the sixth most prevalent cancer worldwide and the fourth most frequent cause of cancer-related deaths, with about 841,000 new cases and 782,000 deaths in 2018 [1]. In about 80–90% of HCCs, fibrosis, and subsequent cirrhosis is present, thereby being the most significant risk factor [2,3]. In both fibrosis and cirrhosis, a chronic liver inflammation is underlying, which is regulated by a complex network of cytokine-mediated signaling pathways [3,4]. Also, HCC evolves due to metabolic and oxidative stress [5]. The therapy of HCC is challenging due to its late diagnosis, mostly at an advanced stage, its chemotherapy-resistant nature, and limited treatment options [3]. Therefore, there is a special need for research in diagnostic and prognostic biomarkers and therapeutic targets for HCC [6,7]. Promising potential therapeutic targets are long non-coding RNAs (lncRNAs), which combine several benefits due to their participation in different cellular complexes [7]. In the context of HCC, the lncRNA *H19* has been a matter of prolonged debate. The biological functions of *H19* in hepatocarcinogenesis remain controversial. It has been a major challenge to interpret the data on *H19* expression in HCC given the small number of samples in most of the available studies and the different experimental settings [5]. Therefore, this review aimed at summarizing the different studies and settings in order to provide an overview of the lncRNA *H19*, which might represent a therapeutic target in HCC.

## 2. Hepatocellular Carcinoma

Our liver can regenerate in both volume and function after chemical or physical damage [8]. But malignant tumors, such as hepatocellular carcinoma (HCC), which mostly develops from chronic liver disease (70–90% of all patients), pose a challenge to the renewal of the liver [9,10]. HCC is a frequent and deadly tumor due to metastasis and recurrence in 50–70% of patients five years post-surgery [1,3,9,10].

The limited treatment options available range from chemoembolization therapy to systemic therapy. Sorafenib, as an inhibitor of tumor cell proliferation and angiogenesis [11,12] is effective in first-line therapy. It was the first drug, which demonstrated a survival benefit in patients with advanced HCC [12]. Other kinase inhibitors, such as regorafenib, that blocks the activity of protein kinases involved in angiogenesis, oncogenesis, and the tumor microenvironment, are used in second-line therapy [12]. Clinical trials in phase III, for first-line and second-line treatments, and combinations with sorafenib, are already completed but do not point towards an exceptional improvement of HCC therapy. The treatment of choice for patients without cirrhosis is liver resection [9,10]. However, systematic chemotherapy and chemoembolization is ineffective in increasing survival, and resection is appropriate only for a minor patient population, i.e., 5% of patients in the USA and Europe [9]. Therefore, the identification of a biomarker for the early detection of HCC and discovery of improved therapeutic targets could lead to new treatment options.

## 3. The *H19* Locus: Two LncRNAs, a MicroRNA and Two Putative Proteins

**Figure 1:**
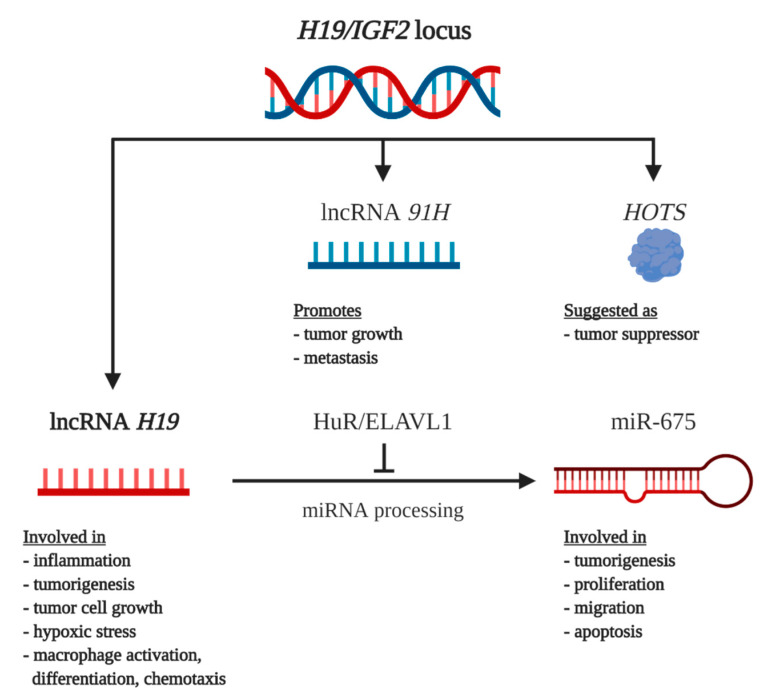
Overview of the transcripts of the lncRNA *H19* locus and their respective functions. Figures created with Biorender.com. HuR/ELAVL1: human antigen R/ELAV-like protein 1; IGF2: Insulin-like growth factor 2

The *H19* locus encodes various transcripts: the main transcript lncRNA *H19*, the encoded microRNA, *miR-675*, and the two antisense transcripts *91H* and H19 opposite tumor suppressor (*HOTS)* [13] (Figure 1).

### 3.1. LncRNA H19

*H19* was originally isolated in murine tissue from different laboratories and was defined as one of the first imprinted genes with maternal expression [14,15,16]. *H19* was classified as a non-coding RNA because of the absence of a detectable translated protein in mice. Furthermore, it has a poor open reading frame (ORF) conservation between mouse and human, but a high overall DNA sequence identity [17]. The gene contains five exons and four small introns and is fully capped, spliced, polyadenylated, and exported into the cytosol [14]. The expression profile of *H19* was first examined during development in the mouse [18]. It is highly expressed during fetal development just as the reciprocally imprinted insulin-like growth factor 2 (*IGF2*) gene. Both genes share the same enhancer elements, which bind and thereby activate the *H19* promotor [13].

*H19* underlies the genomic imprinting: Under imprinted conditions, *H19* is expressed from the maternal allele and *IGF2* from the paternal allele [13,19]. Both genes are controlled by a differentially methylated region (DMR), which is located in an intergenic region of *IGF2* [20]. In the imprinted state, the DMR on the maternal allele is unmethylated so that the zinc finger protein CCCTC-binding factor (CTCF) can bind, thereby insulating and preventing the expression of *IGF2*. On the paternal allele, the DMR is methylated, thereby blocking the CTCF insulator activity and enabling *IGF2* expression. Further, monoallelic expression of *H19* and *IGF2* is regulated in two distinct ways by a shared cis-acting element [21]. Thus, the expression of both genes is tightly coordinated, mostly in endoderm and mesoderm derived tissues during fetal life [19]. The regulation of the imprinting control region of *H19* has been reviewed by Gabory et al. in more detail [19].

Especially during the development of the fetal liver, *H19* is highly expressed [15,17,22]. It has been shown that *H19* inhibits the proliferation of fetal liver cells and the activity of the Wnt/β-catenin signaling pathway [22]. Interestingly, after birth, the expression of *H19* is strongly down-regulated in all tissues, except for the skeletal muscle [14,23]. *H19* is reactivated in different tumor types, including HCC [24,25,26,27]. As mentioned before, the potential function of *H19* as a tumor-suppressor or as an oncogene is strongly debated [16,24,25,28,29,30,31]. After the downregulation of *H19* in adult livers, the imprinting can be lost in HCC [13]. This so-called loss of imprinting (LOI) is correlated with a hypomethylation, which might be in response to environmental exposures to risk factors for the development of HCC [32,33].

### 3.2. MiR-675

Part of the controversial roles of *H19* observed in tumorigenesis might be explained by independent functions of a microRNA (miRNA) encoded by *H19* [25]. In the fetal liver, *miR-675* is detectable at all stages of embryonic development, although the *H19* transcript is much more abundant [15]. In later life, *miR-675* has various targets like the receptor insulin-like growth factor 1 receptor (*IGF1R*), which is involved in proliferation and migration [5,15] or the tumor suppressor retinoblastoma (*RB*) [34]. It can directly downregulate *IGF-1R*, SMAD family member 1 (*SMAD1*), SMAD family member 5 (*SMAD5*), and cell division cycle 6 (*CDC6*), Cadherin-11, Cadherin-13, *Rb*, runt-related transcription factor 1 (*Runx1*), Nodal Modulator 1, transforming growth factor, beta-induced (*TGFBI*), calcium-binding protein 8 (*CALN1*), and microphthalmia-associated transcription factor (*MITF*) as reviewed by Raveh et al. [25]. Interestingly, in colorectal cancer, *miR-675* promotes carcinogenesis through the downregulation of its target *RB* [34]. However, the RNA-binding protein human antigen R/ELAV-like protein 1 (HuR/ELAVL1) acts as an inhibitor of *miR-675* processing by binding *H19* [5,35]. HuR1/ELAVL1 is significantly upregulated in HCC, whereas the more abundant *miR-675-3p* is downregulated [5]. The less abundant *miR-675-5p* is almost not detectable in HCC [5]. Thus, similar to the controversy regarding the role of *H19* in cancer, there are conflicting opinions about the role of *miR-675* as well [36,37]. However, the contradictory results on *miR-675* might be easily resolved by carefully investigating both miRNAs or by clearly stating which miRNA was analyzed.

Interestingly, *H19* can act as a molecular sponge, i.e., competing endogenous RNA (ceRNA). Thereby, *H19* can affect activity of miRNAs and their respective target mRNA levels [13]. CeRNAs exert widespread regulatory controls by forming ceRNA networks. With this sponge function, *H19* sequesters *let-7*, which targets genes inhibiting the insulin-phosphointositol-3 (PI3K)-mammalian target of rapamycin (mTOR) pathway [13]. In vitro data with chemoresistant cell lines showed higher *H19* and lower *let-7a* levels, and an *H19* knockdown restores chemosensitivity in breast cancer cells [38]. Interestingly, though, a double-negative feedback loop exists between *H19* and *let-7* [39]. Besides the connection with *let-7*, also interactions with other miRNAs are mentioned, such as *miR-200b* and *miRNA-200c* [26,40]. A higher level of complexity can be presumed due to a sponging of *mir-675* by *H19* in ovarian cancer [15,27].

### 3.3. LncRNA 91H

Studies on the *H19* locus have identified an *H19* antisense RNA named *91H* in humans and mice [41]. *91H* is a single 120-kb transcript, which is nuclear and short-lived. It is predominantly expressed from the maternal allele in human and mouse within the *H19* gene region. In HCC tissue and cells, *91H* is overexpressed [42]. High *91H* expression is associated with a poor prognosis in HCC patients: both disease-free survival time and the overall survival time was shorter compared to patients with low *91H* expressions [42]. In vitro studies revealed that a knockdown of *91H* in Hep3B cells down-regulated the expression of insulin-like growth factor 2 (*IGF2)* suggesting a co-regulation of *91H* and *IGF2* [42].

### 3.4. Proteins Encoded at the H19 Locus

In 2011, Onyango and Feinberg showed that the human *H19* locus encodes another maternally expressed translated gene in primates, which is antisense to the *H19* transcript [17]. For this protein, no ORF was found in mice. Overexpression of the so-called H19 opposite tumor suppressor (HOTS) could inhibit choriocarcinoma tumor cell growth. The authors deduced that the protein may mediate tumor suppressor activity through targeting DNA replication and cell cycle regulation [43]. However, this has been the only study describing HOTS protein so far. In 2012, Gascoigne et al. identified an additional H19 sense protein with a size of 26 kDa by an approach integrating transcriptomic and proteomic data [17]. The expression of H19 sense protein was identified only in fetal liver, a myelogenous leukemia cell line (K562), and in testes [17], while functional analyses of this protein have not been performed so far.

As mentioned above, *H19* as functionally-validated lncRNA has a poor ORF of 256 nucleotides. But compared to the definition of lncRNAs with a threshold set to 300 nucleotides, *H19* has a large ORF. Therefore, it is impossible to exclude the possibility that *H19* might be translated into functional proteins, perhaps in cell-type-specific contexts or at low levels [44].

## 4. The Role of *H19* in Pre-Malignant Liver Disease

Hepatocarcinogenesis usually evolves after years of chronic inflammatory liver disease. Non-alcoholic fatty liver disease (NAFLD) is the most common liver disease and is expected to be the top indication for liver transplantation within the next decade. NAFLD is characterized by the accumulation of lipids in the liver. This so-called steatosis can progress to an inflammatory disease, which is then termed non-alcoholic steatohepatitis (NASH). NASH patients, as well as alcoholic steatohepatitis (ASH) patients, may develop liver fibrosis leading to cirrhosis, which is the main risk factor for hepatocarcinogenesis. The role of *H19* in hepatic function and liver diseases has been comprehensively reviewed by Pope et al. [13]. Thus, we will focus on the more recent achievements in the following chapter.

The co-regulated gene *IGF2* has been shown to induce hepatic lipid deposition [45]. Further, transgenic overexpression of the *IGF2* mRNA binding protein (*IGF2BP2/IMP2*), leading to upregulated expression of *H19*, induces steatosis [46] and promotes NASH progression [47,48]. However, the role of *H19* in this disease context had remained elusive until a very recent study by Wang and colleagues. These authors observed increased expression of *H19* in steatosis and high-fat diet (HFD)-induced fatty liver [49]. *H19* has been reported to induce numerous genes involved in lipid metabolism, whereby Carbohydrate-responsive element-binding protein (ChREBP) is suggested to be mechanistically important [49]. *H19* overexpression up-regulates mammalian Target of Rapamycin (mTOR) complex 1 (mTORC1) signaling complex in hepatocytes, which is inhibited by silencing *H19* [49]. *H19* stabilizes the nuclear transcriptional activity of the lipogenic master regulator sterol regulatory element-binding protein 1c (SREBP1c) by interacting with polypyrimidine tract binding protein 1 (PTBP1). Vice versa, *H19* is induced by fatty acids in hepatocytes, and ectopic expression of *H19* induces steatosis [50]. *H19* might further act via its ceRNA function. With this, *H19* was reported to target *miR-130a*, thereby affecting Peroxisome proliferator-activated receptor (PPAR) γ associated lipid deposition [51]. Lipid droplet consumption is based on the complex formation of AMP-activated protein kinase (AMPK) α and LKB1, which is facilitated by *H19* in hepatic stellate cells (HSCs) [52]. In contrast, after decreasing hepatic triglyceride content by inhibition of perilipin 2 (PLIN2), which belongs to the lipid droplet (LD) associated proteins and correlates with the amount of LDs [53], *H19* expression is highly altered [54]. In line with the latter study, *H19* is downregulated in livers of db/db mice [55].

The progression of NASH is marked by hepatic fibrosis, the scarring of the liver. Fibrosis occurs due to increased deposition of extracellular matrix in the hepatic tissue. Whole transcriptome RNA-sequencing analysis revealed *H19* to be altered in cirrhotic livers compared to normal healthy tissue [56]. Furthermore, *H19* is increased in the classical mouse model of liver fibrosis, treatment with CCl_4_, and seems to play a role, especially in cholestatic liver fibrosis. In the latter condition, knockdown of *H19* still partially rescues B-cell lymphoma 2 (BCL2)-induced liver injury [57]. In that case, *H19* prevents the inhibition of epithelial cell adhesion molecule (EpCAM) by zinc finger E-box-binding homeobox 1 (ZEB1) through repression of promoter activity [58].

Hepatic stellate cells (HSCs) are the predominant cell type producing extracellular matrix, and their activation promotes fibrogenesis. Activated HSCs show an induction of *miR-874* [59], which can be sequestered by *H19* [60]. Another miRNA affected by *H19* is *miR-148a*. *MiR-148a* targets the protease of ubiquitin specific protease 4 (*USP4)*, which stabilizes transforming growth factor beta (TGF) β receptor 1. Thus, *H19* promotes TGFβ signaling through the *miR-148a*/USP4 axis in both HSCs and hepatocytes [61]. *H19* was reported to be negatively associated with phosphorylation of extracellular signal-regulated kinases 1/2 (ERK1/2) in HSCs [62]. This might be in part due to the repression of IGF1R and, thus, suppression of HSC proliferation [63].

Due to its multicellular composition of hepatocytes, liver sinusoidal endothelial cells, Kupffer cells, lymphocytes, biliary cells, and HSCs, the liver needs a complex intercellular communication network. Besides autocrine, paracrine, endocrine, and cell-cell contacts, exosomes have recently gained attention as an additional mechanism of cellular communication. Therefore, exosomes contain various bioactive molecules, such as lipids, proteins, RNA, and DNA. Interestingly, serum exosomes from human patients with cirrhosis contain elevated *H19* levels [29]. In *Mdr2^−/−^* mice and CCl_4_ treated mice, *H19* from cholangiocyte derived exosomes promotes cholestatic liver injury and correlates with disease progression [50]. The cholangiocyte-derived exosomal *H19* can induce the expression and secretion of chemokine ligand 2 (CCL2) and interleukin 6 (IL6) in Kupffer cells. Further, it is associated with M1 polarization of Kupffer cells and the recruitment and differentiation of bone marrow-derived macrophages [64].

Taken together, *H19* plays a role in premalignant stages of liver disease. Although, some observations are controversial, most of the studies point towards a disease-promoting action of *H19* in this context. Still, only a few potential mechanisms shedding light on the role of *H19* in premalignant liver diseases are described so far. Future studies investigating the exact mechanisms by which *H19* affects the development of simple steatosis and NASH progression towards fibrosis will help to assess *H19*’s role in the early steps of hepatocarcinogenesis.

## 5. The Role of *H19* in HCC

Conclusions from studies in the literature range from *H19* being a tumor suppressor to *H19* being an oncogene in HCC (Table 1, Table 2 and Table 3). At least part of these contradictory results might be due to the diversity of the models used as well as small sample sizes in studies using human patient samples. In order to shed light on this controversial issue, we will discuss the experimental design of several studies in more detail in the following section.

### 5.1. The Role of H19 In Vitro

Supporting the role of *H19* as an oncogene, *H19* was shown to be upregulated in the liver cancer cell line Hep3B as a response to hypoxic stress [24]. Hep3B cells were transfected with siRNA directed against *H19*. To create hypoxic conditions, cells were placed into an Aneoropack rectangular jar under hypoxic conditions [24]. These experiments were not confirmed in additional cell lines. In hypoxic stress conditions, *H19* was significantly upregulated in the control cells compared to the *H19* siRNA treated cells. Later on, these results were confirmed in a follow-up study [65], in which Matouk et al. investigated over nine HCC cell lines, e.g., HepG2, Huh7, HepB3, under hypoxic conditions. Further, in the presence of the tumor suppressor p53, elevation of *H19* is inhibited under hypoxic stress [65]. Consistently, also in normoxic conditions *H19* shRNA knockdown inhibits migration and invasion in HepG2 cells and Bel-7402 cells, which can be rescued by a miR-15 inhibitor [66]. In doxorubicin-resistant R-HepG2 cells an eight-fold upregulation of *H19* was observed, accompanied with an overexpression of the multi-drug resistant 1 (MDR1) gene and a decreased accumulation of doxorubicin [67]. Knockdown of *H19* in R-HepG2 cells suppresses *MDR1* expression, increased cellular doxorubicin accumulation level, and sensitizes towards doxorubicin toxicity [67]. These results shows that *H19* might induce the MDR1-associated drug resistance in liver cancer cells. In contrast, Schultheiss et al. investigated the function of *H19* in HCC in vitro and in vivo and observed opposite results compared to the latter studies [5]. The study determined the role of *H19* in three different human liver cancer cell lines (HepG2, Plc/Prf5, and Huh7) resistant to either doxorubicin or sorafenib. *H19* was down-regulated in all six chemoresistant cell lines [5]. Regarding chemosensitivity, liver cancer cells stably overexpressing *H19,* established by plasmid transfection, were treated with doxorubicin or sorafenib. *H19* overexpression decreases colonoy formation, indicating a tumor cell survival suppressing and chemotherapy-sensitizing action of *H19* [5]. *H19*-induced apoptosis in HepG2 cells is regulated by autophagy through the phosphoinositol-3 kinase (PI3K) signaling pathway [68]. Interestingly, under chemotherapy, human HCC cell lines showed a significantly elevated cell survival and proliferation after an *H19* knockdown [69]. Therefore, Ma et al. examined different human HCC cell lines (HepG2, Hep3B, Bel-7402, QGY-7703, SMMC-7721) when exposed to radiation and chemotherapies (e.g., docetaxel, paclitaxel) [69]. The study shows that after knockdown of *H19* and treatment with chemotherapeutics, cell survival significantly increases compared to the control, especially in Bel-7402 and QGY-7703 cells. In addition, in Bel-7402 cells, the apoptosis rate decreases [69] again supporting the idea that *H19* works as a tumor suppressor. Furthermore, overexpression of *H19* in SMMC-7721 cells inhibits tumor cell invasion by the reversion of EMT [70]. However, any conclusions drawn from experiments involving Bel-7402, QGY-7703, SMMC-7721 as well as MHCC97L, Bel-7404, QGY-7701, and QSG-7701 are problematic due to a contamination with HeLa cells [71]. SKHEP1 cells were reported to be of endothelial origin [72]. The conflicting results could simply reflect the fact that the studies did not use the same conditions: different cell lines and also different methods were used. The Hep3B cell line might be more suitable compared to HepG2 cells for studies in HCC since Hep3B cells are tumorigenic in contrast to HepG2 cells [73]. Furthermore, it should be considered that Hep3B and HepG2 have differences in ethnic origin, biology, genetics and biochemistry, which leads to e.g., different chemosensitivity to therapeutic drugs [73]. Still, tumor suppressive action was observed in three (Schultheiss et al.) and at least two (Ma et al.) different liver cancer cell lines [5,69]. In summary, the results of all these studies are indeed contradictory, confirming that different cell culture systems can have a huge impact on the outcome of the study. Thus, one has to carefully choose the right model for the respective underlying hypothesis. Future studies should also use 3D cell culture, which is more appropriate than 2D cell culture in terms of tumor research. The selection of the specific cell line should be based on the aim of the respective study, e.g., whether cells are expected be derived from an Hepatitis B virus (HBV)-associated tumor, should be alpha-fetoprotein (AFP) positive, or need to express specific gene signatures. In general, cell lines largely retain the genomic and transcriptomic features of primary HCCs even with an increasing number of cell passages [74]. However, there is a high variation of correlation of gene expression among the commonly used HCC cell lines and HCC tissue from the TCGA data set [75]. Thus, for general questions, the best correlated cell lines, i.e., HepG2, C3A, HuH1, Jhh5, SNU886, SNU761, SNU878, LI7, PLC/PRF75, HepB, and JHH7 should be used. Regarding gene signatures, HCC is a very heterogeneous tumor with multiple molecular subtypes [76,77,78,79,80,81,82]. If a specific subtype is investigated, the study by Hirschfield et al. may be helpful for cell line selection, especially with regard to drug response [83].

### 5.2. The Role of H19 In Vivo—Animal Model

In vivo models also clearly show involvement of H19 in HCC. In addition to the in vitro data, Matouk et al. investigated tumor growth in vivo in CD-1 nude mice by subcutaneously injecting *H19* knockdown Hep3B cells into the dorsal flank of athymic female CD-1 nude mice [24]. *H19* knockdown significantly inhibited tumor growth, and, in some cases, tumors did not form at all, suggesting an oncogenic role for *H19* in HCC. In contrast, Schultheiss et al. examined the role of *H19* in a hepatocarcinogenesis model [5]. *H19* knockout mice were injected with 5 mg/kg body weight diethylnitrosamine (DEN), an inflammation-inducing carcinogen, and sacrificed 24 weeks after injection [5]. *H19* knockout leads to increased tumor development and tumor multiplicity upon DEN, thus showing a tumor-suppressive effect of *H19* in vivo and supporting their previous conclusions from in vitro models. Interestingly, this was independent of the reciprocally imprinted *IGF2*. Another *H19* knockout mouse model study from Yoshimizu et al. used a transgenic SV40 hepatocarcinoma model [16]. Males of this transgenic line have a low but constitutive expression of the SV40 T antigen, which eventually leads to the development of multiple hepatocellular carcinomas. The lack of *H19* leads to acceleration in the development of liver tumors and larger tumors [16]. In line with the study from Schultheiss et al., the results were independent of *IGF2* levels [16].

Further, support for the hypothesis that *H19* is a tumor suppressor was obtained from a xenograft study by Zhang et al. [70]. In this case, the mice received a subcutaneous injection of HCCLM3 cells into both flanks. In mice treated with sh*H19* HCCLM3 cells, the number of intrahepatic tumor metastases significantly increased [70].

Regarding the latter four studies, *H19* appears to function as a tumor suppressor rather than an oncogene in hepatocarcinogenesis. The main differences between these studies are again in the conditions of the experiments. Matouk et al. used a xenograft model to investigate their hypothesis [24]. One has to keep in mind that xenograft models are not suitable to study carcinogenesis, but rather tumor progression as analyzed by Zhang et al. [70]. For tumor development, a specific microenvironment, including an intact immune system, is required [84]. Furthermore, the immunodeficient mouse model is not able to build an inflammatory environment with an intact immune system, which is typical in the human setting of HCC development [84].

Another interesting aspect was highlighted by the study by Ramani et al. [85]. Mice lacking Prohibitin 1 (Phb1), a mitochondrial chaperone protein, which regulates downstream signaling pathways, apoptosis, and transcriptional activation, in exhibit severe oxidative stress, fibrosis, and liver cancer. *H19* and *IGF2* are significantly upregulated in Phb1 deficient livers [85]. However, it is unclear whether the induction of *H19* or *IGF2* by *Phb1* knockout leads to tumor development.

Taken together, most in vivo studies support a tumor suppressor function for *H19* [5,16,70], with the exception of one xenograft model [24].

### 5.3. The Role of H19 Ex Vivo—Human Samples

In vivo models suggest that *H19* is a tumor suppressor in hepatocarcinogenesis rather than an oncogene. Nevertheless, it is essential to compare these results with human HCC samples. Most human studies show that *H19* is overexpressed in HCC [28,30,66,86,87,88,89,90,91]. Early investigations indicated an elevation of *H19* in human HCC samples [32,89,90,91]. However, it should be noted that these studies used small sample sizes (*n* = 18–80) as well as different methods, such as in situ hybridization (ISH) or qPCR. Besides, *H19* was not expressed in all of the analyzed HCC samples. Therefore, the number of samples should have been even higher in order to achieve statistical significance. Yang et al. investigated a cohort with a significant sample size of 240 tumor tissues from primary HCC patients [88]. In this study, *H19* expression correlated with HCC aggressiveness and poor disease outcome. However, the expression of *H19* in healthy tissue was not available in this study. In contrast, Zhang and colleagues described downregulation of *H19* in HCC tissue compared to adjacent non-tumorous tissue [70]. The latter study used a unique approach investigating expression of *H19* in different regions of the non-tumorous tissue defined by the distance to the tumor [70]. Interestingly, *H19* expression was highest in the peritumoral area, meaning that the sample collection, especially of the non-tumorous adjacent tissue, has a significant impact on the outcome of the study. Therefore, future studies should be thoroughly conducted regarding sample collection. In line with the findings by Zhang et al. [70], Schultheiss et al. found that the majority of HCC samples show a significantly reduced *H19* expression compared to adjacent non-tumorous tissue [5]. However, in all data sets analyzed by Schultheiss et al., there was a small subgroup of samples showing elevated *H19* expression in tumor tissue. One explanation for these contradictory findings and of specific HCC subgroups with high *H19* levels that show a more aggressive phenotype, has been reported by Iizuka and colleagues [92]. The authors observed that although neither *H19* nor *IGF2* expression levels alone are prognostic, patients with imbalanced, anti-correlated expression levels of both genes are associated with poor survival [92]. Since *H19* and *IGF2* are generally tightly co-regulated, it is important to study bith genes in parallel in future studies. Furthermore, contradictory findings of former studies might be due to different cell composition in sample subsets of the patient cohorts. Interestingly, a recent study showed that tumor-associated macrophages highly express *H19* and thereby promote HCC aggressiveness [93]. Thus, further studies deciphering cell-specific effects of *H19* are needed to clarify this controversy. A promising approach could be single-cell analysis of HCC samples instead of using bulk tissue samples to gain insight into the cell-specifc expression of *H19*.

Wu et al. screened for polymorphisms of *H19* and *IGF2* in HCC samples [32]. They identified three degrees of methylation with hyper- and hypomethylated profiles being strongly associated with an LOI. Biallelic expression of *H19* has been shown for several tumors [94,95], including HCC [24,96]. In general, overexpression of imprinted genes can be induced by either reactivation of the silenced allele resulting in biallelic expression of the gene or higher transcription levels of the active allele. Keeping in mind that HCC is a very heterogeneous tumor entity, this might explain the lack of a difference in the imprinting status in HCC in another study [5]. In the latter study, also the surrounding non-tumorous tissue showed biallelic expression of *H19* [5]. Excitingly, aberrant methylation of the *IGF2* locus in non-tumorous tissue of Hepatitis C virus (HCV) infected livers is predictive for cancer risk or disease progression [97]. It should be emphasized that analysis of imprinting at the *H19/IGF2* locus in the healthy adult liver is complex due to the fact that while *H19* expression is monoallelic, expression of *IGF2* is biallelic [98,99]. In all studies mentioned here, the sample size was quite small. Furthermore, most of the studies do not investigate DNA methylation and the corresponding RNA expression in parallel. Thus, it remains elusive whether LOI of *H19* or *H19* expression independent of the DNA methylation status plays a role in hepatocarcinogenesis. In fact, in colorectal cancer, *IGF2* expression is unrelated to its imprinting status [100], which might be also the case also for *H19*.

Two other exciting studies investigate the association between different *H19* polymorphisms and the susceptibility as well as prognosis on tumorigenesis [86,87]. A study in glioma by Deng et al. with 605 patient samples showed that some polymorphisms decreased susceptibility to glioma, a common and highly fatal brain tumor [86]. The rs3741219 A/G polymorphism, which is located in the first exon of *H19*, is associated with a reduced risk of glioma. Interestingly, this region encodes for the antisense transcript HOTS. But so far, there is no evidence for a co-expression of *H19* and HOTS in vivo [86]. In HCC, the T allele of *H19* rs217727 polymorphism, which is associated with decreased H19 expression, enhanced the survival rate of patients with HCC [101]. However, the study from Wu et al. showed a correlation between *H19* gene polymorphisms and the susceptibility to develop late-stage tumors [87]. Strikingly, two exon-related gene polymorphisms (rs2839689 and rs3741219) and a specific haplotype of *H19* showed an increased susceptibility to HCC. So far, the authors did not find any correlation between *H19* and serological markers of HCC, such as AFP, alanine transaminase (ALT), or aspartate transaminase (AST). Beside the increased risk to develop HCC when carrying the rs2839698 polymorphism, an association with poor prognosis exists in smoking patients [102]. However, the data from the latter studies suggest that different *H19* gene polymorphisms could have either tumor-suppressing or promoting effects. Thus, *H19* polymorphisms might be an explanation for different study outcomes.

## 6. Possible Treatments of HCC with Respect to *H19*

Although the literature is still confusing, the various interactions of the *H19* locus offer many possibilities for novel diagnostics or therapeutic targets in HCC. One possibility was mentioned by Ariel et al. [89]: for the diagnosis of hepatocellular carcinoma in fine-needle aspirates, in situ hybridization (ISH) for *H19* could be performed. They showed that the expression of *H19* was detected with ISH in 13 of 18 samples with HCC. AFP, a commonly used tumor marker in HCC, was found only in nine of 18 samples [89]. Therefore, ISH for *H19* could be a useful diagnostic method.

As mentioned before, *H19* and *IGF2* are often co-expressed in tumor cells. Therefore, Amit et al. examined the therapeutic potential of a double promoter toxin vector H19-DTA-(IGF2)-P4-DTA leading to, expressioned of the diphtheria toxin [103]. The vector had the potential to reach the tumor cells and delivered its toxin intracellularly, without targeting healthy tissues [103]. This would be an innovative approach to specifically target tumor cells.

Another study with a focus on IGF2, but potentially translatable for *H19*, designed a methylated oligonucleotide (MON1) [104]. MON1 is complementary to a region encompassing *IGF2* P4 promoter. It specifically inhibited *IGF2* mRNA accumulation in vitro in Hep3B cells. In vivo, animals bearing engrafted tumors had prolonged survival upon treatment with MON1. Thus, a methylated sense oligonucleotide, complementary to *H19*, may lead to enhanced survival in vivo [104].

Taken together, however, *H19* as a therapeutic target was only investigated under the hypothesis of *H19* being an oncogene. With respect to most of the in vivo and ex vivo studies the tumor suppressor role seems to be more plausible. Of course, re-activation of expression is far more challenging in therapy than inhibiting expression. Still, increasing knowledge about the whole *H19* locus and the transcript-specific functions in HCC could lead to more potential therapeutic strategies for the treatment of HCC or the modulation of the hepatic inflammatory environment in the pre-malignant phase of liver disease.

## 7. Conclusions

The *H19* locus is a complex gene locus and the transcript specific effects have not always been investigated in parallel in the same studies. Data from in vivo models point towards a tumor suppressor effect of *H19* in HCC. Recent human data raise the possibility that *H19* gene polymorphisms and cell-type specific expression could explain many of the contradictory findings in the past. Therefore, a deeper understanding of this gene locus is vital for defining a potential use of *H19* in new diagnostic and therapeutic strategies.

## Figures and Tables

**Table 1:** In vitro studies with the focus on *H19* in hepatocellular carcinoma.

Model	Treatment	Result	Reference
Hep3B	- transfected cells (siRNA) - hypoxic conditions	- *H19* ↓ downregulation of antiapoptotic genes - *H19* ↑ in response to hypoxic stress	[24]
Hep3B, HepG2, Huh7, Plc/Prf5	- transfected cells (p53) - hypoxic conditions	- *H19* ↑ in response to hypoxic stress - p53 inhibits *H19*	[65]
HepG2, SMMC-7721, Bel-7402, Huh7	- transfected cells (shRNA)	- proliferation ↑, migration ↑, invation ↑	[66]
HepG2, R-HepG2	- transfected cells (antisense *H19*) - chemoresistant cells - (doxorubicin, etoposide, vincristine, Taxol) - knockdown of *H19*	- MDR1/P-glycoprotein expression ↓ - cellular doxorubicin accumulation level and sensitized toxicity ↑	[67]
HepG2, Plc/Prf5, Huh7	- *H19* ↑ transfected cells (vector) - chemoresistant cells - (sorafenib, doxorubicin)	- HCC chemoresistance↓, colony formation↓ - *H19* ↓	[5]
HepG2, HCCLM3	- hypoxic conditions	- apoptosis rate ↓, cell damage ↓	[68]
HepG2, Hep3B, QGY-7703	- transfected cells (siRNA)	- survival rates ↑, apoptosis rate ↓	[69]

**Table 2:** In vivo animal studies with the focus on H19 in hepatocellular carcinoma.

Model	Treatment	Result	Reference
CD-1 nude mice	- injection of transfected Hep3B cells (siRNA) in xenograft model	- HCC tumors encountered significant retardation of tumor growth - in some cases tumors did not form at all	[24]
*H19 Δ3* knockout mice	- long-term experiment (24 weeks): intraperitoneally injection of 5 mg/kg body weight diethylnitrosamine (DEN)	- tumor development↑ and tumor cell proliferation↑	[5]
*H19Δ3* knockout mice	–	- tumor development ↑	[16]
Mdr2^−/−^	- bile duct ligation → cholestatic conditions	- cholangiocyte-derived exosomal *H19* correlated with macrophage activation, differentiation, and chemotaxis	[64]
orthotopic liver tumor xenograft model	- injection subcutaneously into both flanks of HCCLM3 cells transfected (shH19)	- smaller tumor volume↓, proliferation rate↓, apoptosis rate↑	[70]
*Phb1* knockout mice	–	- *H19* ↑	[71,85]

**Table 3:** Ex vivo studies with the focus on *H19* in hepatocellular carcinoma.

Cohort Size	Result	Reference
*n* = 39 *n* = 7 controls	- three methylation profiles: hyper-, medium-, and hypomethylated profiles - profiles associated with different imprinting of *IGF2* and *H19*	[32]
*n* = 18	- *H19* expression ↑	[89]
*n* = 27	- 11 samples: no *H19* expression - 16 samples: *H19* ↑	[91]
*n* = 240	- *H19*↑ HCC aggressiveness↑,poor outcome↑	[88]
*n* = 46 *n* = 46 controls	- *H19* expression ↑	[66]
*n* = 60	- *IGF1* positively correlated with *H19*	[92]
*n* = 509	- *H19* ↓	[5]
*n* = 359 *n* = 1190 controls	- gene polymorphisms and specific haplotype of *H19* increased susceptibility to hepatocellular carcinoma	[87]
*n* = 944	- rs2839698 SNP of lncRNA-*H19* was associated with an increased risk of HCC	[102]
*n* = 214	- polymorphism of rs217727 in *H19* was associated with HCC	[101]
